# High mosquito burden and malaria transmission in a district of the city of Douala, Cameroon

**DOI:** 10.1186/1471-2334-12-275

**Published:** 2012-10-30

**Authors:** Christophe Antonio-Nkondjio, Blaise Defo-Talom, Romuald Tagne-Fotso, Billy Tene-Fossog, Cyrille Ndo, Leopold Gustave Lehman, Timoléon Tchuinkam, Pierre Kengne, Parfait Awono-Ambene

**Affiliations:** 1 Laboratoire de Recherche sur le Paludisme, Organisation de Coordination pour la lutte Contre les Endémies en Afrique Centrale (OCEAC), P.O. Box 288, Yaoundé, Cameroun; 2 Vector group, Liverpool School of Tropical Medicine, Pembroke Place, Liverpool, L3 5QA, UK; 3 Faculty of Health Sciences University of Bamenda, P.O. Box 39, Bambili, Cameroon; 4 Faculty of Sciences University of Dschang, P.O Box 067, Dschang, Cameroun; 5 Faculty of Sciences University of Douala, P.O. box 24157, Douala, Cameroon; 6 Faculty of Sciences, University of Yaoundé I, P.O. Box 337, Yaoundé, Cameroon; 7 Institut de Recherche pour le Développement (IRD), UMR MIVEGEC (UM1, UM2, CNRS 5290, IRD 224), 911, avenue Agropolis, P.O. Box 64501, Montpellier cedex 5, 34394, France

**Keywords:** Malaria, Transmission, Anopheles gambiae, Resistance, Douala, Cameroon

## Abstract

**Background:**

Rapid demographic growth in Douala city, Cameroon, has resulted in profound ecological and environmental changes. Although demographic changes can affect anopheline mosquito breeding sites, there is a lack of understanding about the epidemiological impact that such changes might have on vector ecology and malaria transmission.

**Methods:**

A 12-month entomological study was conducted in a highly populated district of Douala called Ndogpassi. Adult mosquitoes were collected using two methods: 1) human landing catches (HLC); and 2) Centers for Disease Control and Prevention (CDC) light traps; these methods were used twice monthly from January to December 2011. Mosquito genus and species were identified with morphological and molecular diagnostic tools. The sampling efficiency of the CDC light trap and HLC were compared. *Anopheles gambiae* infection with *Plasmodium falciparum* was detected using ELISA. Susceptibility to DDT, permethrin, and deltamethrin insecticides were also determined.

**Results:**

A total of 6923 mosquitoes were collected by HLC (5198) and CDC light traps (1725). There was no equivalence in the sampling efficiency between light traps and human landing catches (P > 0.01). With 51% of the total, Culex was the most common, followed by Anopheles (26.14%), Mansonia (22.7%) and Aedes (0.1%). *An. gambiae* ss (M form) comprised ~98% of the total anophelines collected. *An. gambiae* had a biting rate of 0.25 to 49.25 bites per human per night, and was the only species found to be infected with *P. falciparum*. A *P. falciparum* infection rate of 0.5% was calculated (based on enzyme-linked immunosorbent assays using the circumsporozoite surface protein). The entomological inoculation rate was estimated at 31 infective bites per annum. Insecticide susceptibility tests on *An. gambiae* females revealed a mortality rate of 33%, 76% and 98% for DDT, permethrin and deltamethrin, respectively. The West African *kdr* allele (L1014F) was detected in 38 of the 61 *An. gambiae* analyzed (62.3%).

**Conclusions:**

The present study revealed seasonal malaria transmission in Douala. High levels of *An. gambiae* were detected along with a high prevalence of insecticide resistance in this vector population. These findings highlight the need to promote use of insecticide-impregnated bed nets in Douala.

## Background

Urban malaria is an emerging health problem in Africa [1]. Some reports indicate that the rapid demographic growth of some urban areas presents a serious challenge for the control of endemic diseases such as malaria [1,2]. Several factors influence the epidemiology and severity of malaria disease in urban areas; these factors include the geographical characteristics of the town, the level of immunity in the local population, the extent of unplanned urbanization, urban agriculture development, and the degree of human migration from rural to urban areas [2,3]. Although unplanned urbanization characterized by poor housing, lack of sanitation and inadequate surface water drainage is recognized as one of the major factors maintaining the presence of *Anopheles gambiae* in some urban settings [2], it has not been extensively studied in Cameroon.

In Cameroon, malaria affects over 90% of the population and is responsible for 35% of the annual mortality [4]. Despite the regular occurrence of malaria cases in urban settings, only a limited number of studies have addressed the epidemiology of the disease in such settings. Entomological studies in the city of Yaoundé, reported low malaria transmission rates in central districts, but high transmission rates in peripheral locations [5-7]. Parasitological studies on the other hand, revealed an annual prevalence of 35% of *Plasmodium falciparum* asexual parasites carriers, a seasonal variation of parasites prevalence in the population and, a potential infectious reservoir dominated by the age group ranging from 0 – 15 years [8]. The city of Douala, the largest in Cameroon, has, paradoxically, received less attention. Although there are some similarities between Douala and Yaoundé, several climatic and environmental contrasts exist. For example, Douala is a port city close to the Atlantic Ocean with year-round rainfall and a high level of spontaneous urbanization compared with Yaoundé, which is situated inland at high altitude. All this making that Yaoundé and Douala could experience distinctive malaria epidemiological conditions. Since the 1950s, successive changes have been made to the Cameroon National Policy for Malaria Control. From the 1950s to the 1960s malaria control efforts were mainly oriented on indoors residual spraying of insecticides, in the 1980s primary health care services were promoted, and in the 1990s insecticide-treated bed nets came into use. A lack of knowledge about malaria epidemiology in different contexts has undoubtedly contributed to variations in the success of such measures to combat the disease [9]. Thus, better understanding of the factors affecting malaria epidemiology in Douala could assist efforts towards malaria control in the city.

This work falls into a series of studies aimed at assessing the influence of urbanization on malaria vector bionomic and malaria transmission in the two major urban cities of Cameroon: Douala and Yaoundé. A recent study conducted in the city of Douala, revealed the presence of a number of mosquito breeding sites that persisted all year long as well as a high prevalence of mosquito resistance to DDT and permethrin insecticides [10]. Here, we present the results of a 12-month study on the dynamics of malaria transmission and mosquito burden in the district Ndogpassi Douala, which contains hundreds of thousands of urban dwellers.

## Methods

### Study sites

The study took place in Ndogpassi (3° 48’N 10° 08’E), a district of Douala, which is the largest city in Cameroon and comprises around 2.5 million inhabitants [11]. Douala is located within the Congo-Guinean phytogeographic zone near the Atlantic coast and lies about 1 meter above sea level. It has an equatorial climate with two rainy seasons from March to June and August to November and about 3,500 mm of rainfall annually.

The district of Ndogpassi, which a decade ago was situated at the edge of the town, is now fully integrated into the city, thus extending the urban domain. Part of the area is covered by marshland that is exploited during the dry season for urban agriculture. Nearly all the district has numerous stagnant pools of water all year long. Ndogpassi is one of the most densely populated districts in the city with a population estimated to be several hundreds of thousands.

### Adult mosquito collections

Adult mosquitoes were collected from January to December 2011. Two sampling methods were used. (1) Human landing catches (HLC) took place between 19:00 to 06:00. Collections were performed outdoors at four randomly selected sites on two consecutive nights each month (two teams of volunteers collected the mosquitoes each night; one team from 19:00 to 01:00 and the other from 01:00 to 06:00). All volunteers consented to mosquito capture and were given free malaria prophylaxis. (2) Seven light traps (LT) of the Centers for Disease Control and Prevention (CDC) type were placed indoors at seven sites using distances between 50 and 500 m.

The study was conducted under ethical clearance No. 216/CNE/SE/09 from the Cameroon National Ethics Committee Ref IORG0006538-IRB00007847-FWA00016054.

### Field processing of mosquitoes

Mosquitoes were identified at the genus and species level. Anophelines were identified for species type using the morphological characteristics provided by the identification keys of Gillies and Coetzee [12] and Gillies & De Meillon [13]. Specimens were stored individually in numbered tubes containing desiccant, after which they were archived and stored at −20°C until ready for processing in the laboratory at Yaoundé.

### Laboratory processing of anophelines

Members of the *An. gambiae* complex were identified using the molecular diagnostic tools described previously [14]. DNA extracted from a mosquito leg and/or wing was used for analysis. The heads and thoraxes from female anophelines were tested for the presence of the *P. falciparum* circumsporozoite protein (CSP) using enzyme-linked immunosorbent assay (ELISA) [15]. The CSP positive rate was calculated as the ratio of *P. falciparum*-infected mosquitoes over the total number of mosquitoes tested by ELISA. The entomological inoculation rate was calculated by multiplying the human biting rate estimated from HLC by the CSP rate.

### Insecticide susceptibility tests

Insecticide susceptibility tests were performed on 2- to 4-day-old unfed *An. gambiae* s.l. The *An. gambiae* larvae used for this analysis were collected from temporary and semi-permanent breeding sites situated near the study area. Batches of 20 to 25 mosquitoes per tube were exposed to insecticide-impregnated papers (Liverpool School of Tropical Medicine, UK) for 1 hour. The insecticide susceptible *An. gambiae* Kisumu strain was used as a control to measure the effectiveness of the impregnated papers. The following diagnostic concentrations of insecticides were tested: 4% DDT, 0.75% permethrin, and 0.05% deltamethrin. The numbers of mosquitoes knocked down by the insecticide were recorded every 5 minutes during exposure. After exposure, mosquitoes were fed with a 10% glucose solution and the number of dead mosquitoes was recorded 24 hours post-exposure. Tests using untreated papers were systematically run as controls. The mortality rates were corrected using the Abbot formula [16] whenever the mortality rate of the controls was between 5 and 20%. World Health Organization criteria [17] were used to evaluate the resistance and susceptibility status of the mosquito population tested. Three classes of insecticide susceptibility were defined: insecticide resistant (<80%), insecticide tolerant (80 to 97%), and insecticide susceptible (>97%).

To screen for the presence of *kdr* alleles (L1014F and L1014S) that confer resistance to DDT and pyrethroid, DNA extracted from individuals exposed to insecticides was tested using the Hot Ligation Oligonucleotide Assay from Lynd et al. [18].

### Data analyses

Houses were selected for light trap catches where the number of occupants comprised 8 to 12 individuals; this approach reduced bias from large differences in the number of human inhabitants per site. Prior to the study, preliminary mosquito collections were conducted using the light traps. Only houses with the highest numbers of mosquitoes were selected for further investigation. To assess linear correlations between the two collection methods, the Pearson correlation coefficient was used to calculate the average number of mosquitoes collected nightly by the LT and HLC methods. Prior to analysis, the average number for each catch (x) was transformed to Y = log(x + 1). To compare methods and determine if mosquito abundance was affected by the sampling efficiency of each method used, the ratio of the number of mosquitoes in LT to the number of mosquitoes in HLC (Log(HLC + 1)-Log(LT + 1)) was plotted against the average abundance [Log(HLC + 1) + Log(LT + 1)]/2 as described by Overgaard et al. [19].

## Results

### Field sampling

A total of 6923 mosquitoes belonging to four genera were collected; these comprised Anopheles, Culex, Mansonia, and Aedes. Of these 6923 mosquitoes, 5198 were collected by a total of 96 men-night catches while 1725 were collected over 168 nights using CDC LT. Culex (51% of the total) was the most prevalent species, followed by Anopheles (26.14%), Mansonia (22.7%) and Aedes (0.1%) (Table 1). The majority of the Culex species were *Culex quinquefasciatus*, whereas Aedes species comprised *A. aegypti* and *A. albopictus*. Two species of anophelines, namely, *An. gambiae* sensu stricto (1805/1810) and *An. ziemanni* (5/1810) were collected. For molecular identification of the *An. gambiae* M and S forms, an average of 20 to 40 mosquitoes were randomly selected from those collected each month using both HLC and CDC LT methods. The results of these collections showed that *An. gambiae* specimens mostly comprised the M molecular form (384/386); only two specimens were of the S molecular form (2/386).

**Table 1:** Number of mosquitoes collected in Ndogpassi district, Douala, using CDC light traps and night landing catches (January to December 2011)

**Species**	**Night landing catches**		**CDC light traps**		**Total**	
	**N**	**B/m/n**	**N**	**Trap/N**	**N**	**%**
Aedes	2	0.04	5	0.03	7	0.10%
Culex	2058	44.74	1474	8.77	3532	51.02%
Mansonia	1449	31.50	125	0.74	1574	22.74%
*An. gambiae*	1685	36.63	120	0.71	1805	26.07%
*An. ziemanni*	4	0.08	1	0.01	5	0.07%
Total	5198	113	1725	10.27	6923	

N : number of mosquitoes collected, B/m/n : Bites/man/night, Trap/N : number collected in a trap per night.

**Figure 1:**
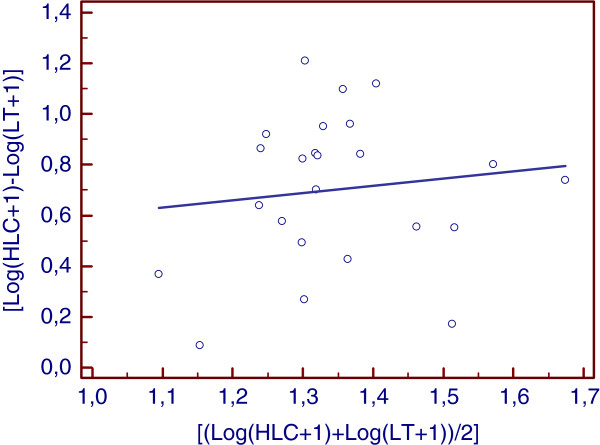
Relation between light trap catches (LT), human landing catches (HLC) and vector abundance.

The average number of mosquitoes collected by HLC was 54.2 mosquitoes per man per night, whereas the average number of mosquitoes collected by CDC LT was 10.3 mosquitoes per trap per night. Culex mosquitoes were most frequently collected by light traps (41.7% of the total) compared with *An. gambiae* and Mansonia (6.6% and 7.9% of their total numbers, respectively). The Pearson correlation coefficient, which was used to assess the relationship between nightly LT and HLC catches, indicated that there was no consistency in the sampling efficiency between LT and HLC when all mosquitoes were considered (r = −0.35, P = 0.53). When mosquitoes were separated into the three most common genera, the correlation was positive but not significant for Anopheles (r = 0.44, P = 0.03) and Culex ( r = 0.36, P = 0.09) but negative for Mansonia (r = −0.16, P = 0,46). Figure 1 shows an analysis of the ratio of LT to HLC plotted against mosquito abundance. No significant tendency for increased sampling effect with increasing mosquito abundance was detected (P = 0.53) (Figure 1).

### Biting rates

**Figure 2:**
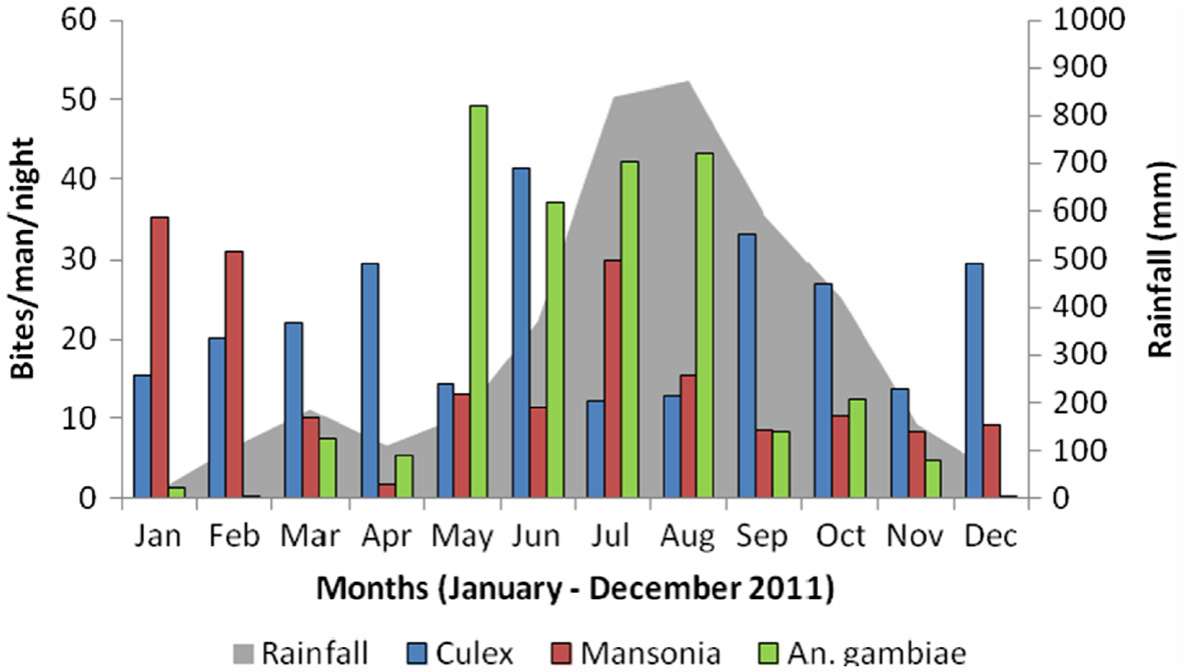
**Graph showing the biting rates for culicines and *Anopheles gambiae *in Ndogpassi district, Douala, from January to December 2011.**

The mosquito burden in Ndogpassi was mainly associated with the presence of *An. gambiae*, Culex and Mansonia. These mosquitoes were present all year long with biting rates varying with the rainfall. *An. gambiae* was responsible for a biting rate of 0.25–49.3 bites per man per night. Culex mosquitoes were present all year long, and their biting rates always exceeded 10 bites per man per night. In contrast, Mansonia were more common during the long dry season in January and February (Figure 2). The following seasonal variations in *An. gambiae* numbers were recorded (in bites per man per night): 42.12 and 1.87 during the short (July) and long dry seasons (December to February), respectively, and 99.13 and 68.63 during the short (March–June) and long (August–November) rainy seasons, respectively.

**Figure 3:**
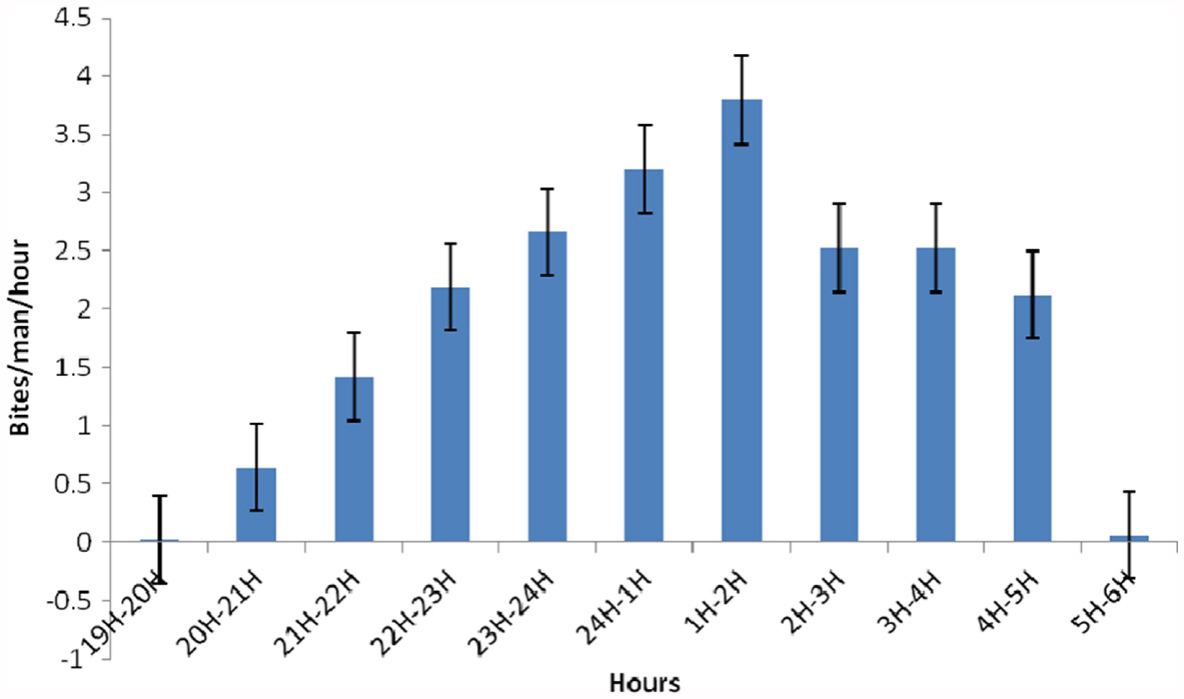
**Night biting cycle of *An. gambiae *in the district of Ndogpassi Douala.**

Figure 3 shows the cycle of night-time biting activity where it can be seen that *An. gambiae* densities were higher between 10 PM and 5 AM with a peak biting activity between 1 and 2 AM (Figure 3).

### Malaria transmission and infection rates

**Figure 4:**
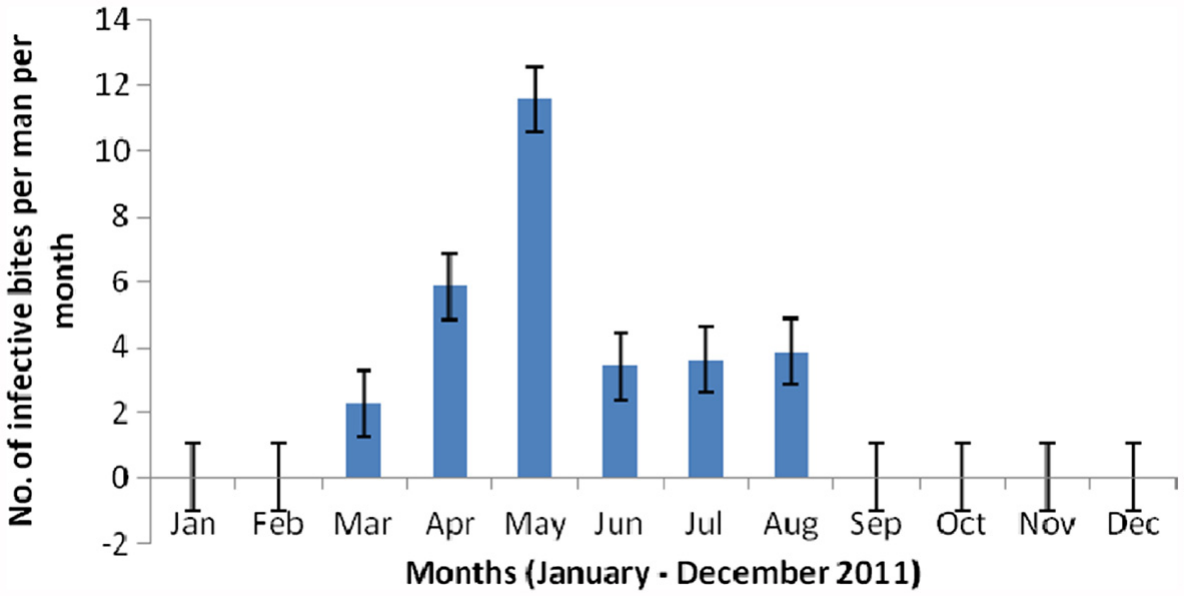
Monthly malaria transmission in Ndogpassi, Douala from January to December 2011.

Nine *An. gambiae* from the 1765 anophelines tested by ELISA (4 *An. ziemanni* and 1761 *An. gambiae*) were found to be infected with *P. falciparum*. The *P. falciparum* infection rate, which was calculated from the number of positive CSP ELISA results, was 0.5% (95% CI: 0.2% - 0.97%). One of the infected mosquitoes was collected by CDC LT, while the rest were collected by HLC. We found that malaria transmission was seasonal and occurred from March to August, with the highest transmission recorded in May 2011 (Figure 4). The annual entomological inoculation rate was estimated at 31 infective bites.

### Insecticide susceptibility assays

A total of 757 *An. gambiae* females were tested for their susceptibility to DDT and pyrethroid insecticides. Mortality rates of 33%, 76% and 98% were recorded for DDT, permethrin and deltamethrin, respectively. The *An. gambiae* insecticide susceptible strain Kisumu, which was used as a control, had a mortality rate of 100% for permethrin (100/100) and deltamethrin (100/100), and 94% for 4% DDT (94/100). Interestingly, we noted that the mosquito knockdown times were four to 12 times higher than the corresponding values for the Kisumu strain (Table 2). *kdr* allele screening was conducted on 61 specimens selected from mosquitoes that survived exposure to DDT, permethrin and deltamethrin; 38 (62.3%) were found to carry the L1014F allele at this locus. In contrast, the L1014S allele, which is common in East Africa, was not detected. Twenty-seven samples were homozygous for the L1014 F allele, whereas 11 samples were heterozygous at this locus.

**Table 2:** **Knockdown times and mortality rates for *Anopheles gambiae *field populations in Ndogpassi, Douala, after exposure to 4% DDT, 0.75% permethrin or 0.05% deltamethrin**

**Insecticide**	**N**	**Knockdown times Kdt50 (95% CI) Min**	**Kdt95 (95% CI) Min**	**KdT50R**	**Mortality**
4% DDT	249	226.5 (0.27 – 1849)	520.5 (0.01 – 25442)	12.04	**33%**
0.75% permethrin	350	110.7 (86.6 – 175)	389.8 (227.6 – 1094)	12	**76%**
0.05% deltamethrin	158	44.3 (42 – 47.02)	108.6 (94.9 – 129.7)	4.7	98%

N: number of mosquitoes tested, Kd: knockdown times, Bold: resistant populations.

Kdt50R: kdT50 of tested population divided by KdT50 of Kisumu strain.

KdT50: Kd time in minutes for 50% mosquitoes.

KdT95: Kd time in minute for 95% mosquitoes.

Mortality: mortality rate 24 hours post exposure to the insecticide.

## Discussion

Our data indicate that frequent malaria transmission occurs in Douala city, a finding that is consistent with the majority of studies conducted in urban settings within sub-Saharan Africa [20-25]. The mosquito burden was primarily Culex, followed by *A. gambiae*, and Mansonia. The large number of Culex suggests that the area is experiencing difficulties with water and waste management of the type that is common to unplanned urbanized areas in Africa [26,27]. The *An. gambiae* densities were far greater than what might have been expected, thus supporting the possibility of adaptation of this species to urban areas [28]. Although the *An. gambiae* biting densities were subject to seasonal fluctuations, this species was present all year long owing to the presence of abundant collections of standing water in the district. However, *An. gambiae* samples consisted almost exclusively of the M form, a finding consistent with earlier studies conducted in Cameroon that indicated the high prevalence of this molecular form in coastal areas [29-31]. The predominance of the M form over the S form in the study area is most likely associated with its higher tolerance to salinity (Costantini, unpublished data).

Human landing catches appeared to be far more efficient than CDC light traps. This observation is consistent with data from several studies comparing the efficacy of HLC with a diverse range of mosquito collection methods [32,33]. Indeed during HLC, mosquitoes are attracted by both visual and chemical stimuli, whereas CDC light traps use only visual stimuli. However, the fact that HLC was undertaken outdoors while CDC LT was conducted indoors could have overestimated the efficiency of HLC compared with CDC LT. Because of the personal and property related security issues in Douala, household owners did not permit us to conduct indoor HLC; this prompted us to adjust the study design, whereby HLC was conducted exclusively outdoors while CDC LT continued to be placed indoors. The number of species collected fluctuated substantially between the two methods but was in agreement with previous studies that have indicated that the efficiency of a collection method can vary according to the composition of the mosquito species present, mosquito densities, availability of alternative hosts, and city lighting [33-35]. The sampling efficiency of both collection methods was found to be independent of vector abundance. Although comparisons between collection methods commonly use log transformation of direct counts, the method used here would have little bias because it uses log(x + 1) instead of log(x) [19]. However, use of negative binomial regression analysis or Bayesian estimations might be more appropriate for some studies [19].

Malaria transmission estimates in Ndogpassi district were relatively high compared to records from similar urban settings [27,36,37]. The data are consistent with parasitological and clinical records revealing a high prevalence of malarial disease in the population, however [38]. Malaria transmission was detected only during the rainy season, despite the continuous presence of *An. gambiae* and could probably be associated to the low infectiousness of the human reservoir [8,21,36,39] alternatively, it could reflect the influence of high temperature and low humidity on the longevity and vectorial capacity of *An. gambiae* during the dry season. Several factors can influence parasite prevalence in humans and mosquito infectivity; these include, for example, the use of protective measures against mosquitoes, health seeking behaviors, socio-economic status, and immunity [40-42].

Although malaria transmission estimates were not assessed across the city, an earlier study suggested that there might be a heterogeneous risk for malaria transmission that is dependent on the distribution of *An. gambiae*[10]. The situation in Douala could be similar to that of Yaoundé where malaria transmission has been estimated at 277–365 infectious bites per human per year in the peripheral districts, and 3–33 infectious bites per human per year in the central districts [7,8,27]. According to Robert et al. [37], the annual inoculation rates in sub-Saharan Africa could be as high as 7.1 in the city centers, 45.8 in peri-urban areas and 167.7 in rural areas. The data from this study exceeded the anticipated estimates for central districts and could indicate that the high malaria prevalence is maintained by frequent migration of populations from highly endemic rural areas to the city. Moreover, the fact that no transmission was detected during certain periods of the year does not totally exclude the possibility of perennial malaria transmission since clinical cases were reported all year long in the local health care center (data not shown). Clearly, further studies for are needed to obtain better understanding of all the factors influencing malaria transmission in Douala.

The level of *An. gambiae* susceptibility to DDT and pyrethroid insecticides, and the frequency of the *kdr* allele in the *An. gambiae* population were in agreement with previous studies [10,43]. The high prevalence of insecticide resistance in the mosquito population of Ndogpassi district can be attributed to the frequent use of chemical insecticides for market gardening and personal protection [44]. Apart from insecticide-treated bed nets, insecticide sprays and coils were reported to be in regular use in households [44], whereas up to eight different insecticide mixtures including pyrethroids, organochlorines, organophosphorus and carbamates have also been reported to be in regular use for urban farming in Cameroon [45]. A direct association between urban farming and increased insecticide resistance in malaria vectors has been reported in several studies [23,25,45,46]. Despite the high prevalence of the *kdr* allele in mosquito populations, and its close association with resistance to both permethrin and DDT [45], this resistance mechanism might not be fully responsible for all of the insecticide resistance phenotypes that exist. Further studies are underway to explore the importance of metabolic resistance to insecticides in mosquito populations. The rapid expansion of insecticide resistance is becoming a serious challenge for malaria control across Africa that calls for sustainable solutions [47,48].

## Conclusions

The present study reveals high densities of *An. gambiae* associated with seasonal malaria transmission in the district of Ndogpassi, Douala, Cameroon. Moreover, our surveys showed a high prevalence of *An. gambiae* that are resistant to both DDT and permethrin. These data might be of great importance to assess the dynamic of malaria transmission and insecticide resistance in Douala after the recent nationwide free distribution of long lasting insecticidal nets to the population.

## Competing interests

The authors declare that they have no competing interests.

## Authors’ contributions

CAN, PAA conceived of and designed the study protocol. BDT, RTF, PK, BTF, CN, PAA, and CAN participated in the field sample collections. BDT, BTF, CN, and CAN carried out the molecular and ELISA analyses; LGL, TT, and PK helped to draft and revise the manuscript. CAN was responsible for data analysis and interpretation and also wrote the paper. All authors have read and approved the final version.

## Pre-publication history

The pre-publication history for this paper can be accessed here:

http://www.biomedcentral.com/1471-2334/12/275/prepub

## References

[B1] Hay S Guerra C Tatem A Atkinson P Snow R Urbanization, malaria transmission and disease burden in Africa Nat Rev Microbiol 2005 3 81 90 10.1038/nrmicro1069 15608702 PMC3130901

[B2] Keiser J Utzinger J Caldas De Castro M Smith T Tanner M Singer B Urbanization in sub-saharan Africa and implication for malaria control AmJTrop Med Hyg 2004 71 118 127 15331827

[B3] Wang S Lengeler C Smith T Vounatsou P Cisse G Diallo D Akogbeto M Mtasiwa D Teklehaimanot A Tanner M Rapid urban malaria appraisal (RUMA) in sub-Saharan Africa Malar J 2005 4 40 10.1186/1475-2875-4-40 16153298 PMC1249588

[B4] Minsanté Cameroun: vers un meilleur accès à la prévention et au traitement du SIDA, de la tuberculose et du paludisme 2008 http://wwwafriscoopnet/journal/spipphp

[B5] Fondjo E Robert V Le Goff G Toto J Carnevale P Urban malaria transmission in Yaounde (Cameroon). 2. Entomologic study in 2 semi urban districts Bull Soc Path Exot 1992 85 57 63 1596961

[B6] Nimpaye H Van Der Kolk M Fontenille D Boudin C Le paludisme urbain à Yaoundé (Cameroun) en 2000. Etude entomologique dans le quartier central «Dakar» Bull Liais Doc OCEAC 2001 34 11 14

[B7] Antonio-Nkondjio C Awono-Ambene H Toto J Meunier J Zebaze-Kemleu S Nyambam R Wondji C Tchuinkam T Fontenille D High malaria transmission intensity in sub-urban area of Yaounde: the capital city of Cameroon J Med Entomol 2002 39 350 355 10.1603/0022-2585-39.2.350 11931035

[B8] Van der Kolk M Etti Tebo A Nimpaye H Ngo Ndombol D Sauerwein R Eling W Transmission of Plasmodium falciparum in urban Yaoundé Cameroon is seasonal and age-dependent Trans R Soc Trop Med Hyg 2003 97 375 379 10.1016/S0035-9203(03)90059-9 15259460

[B9] Carnevale P Mouchet J La lutte antivectorielle au Cameroun. Passé-Présent-Avenir. Réflexions Entomol Méd 2001 2181 202 208 16579079

[B10] Antonio-Nkondjio C Tene-Fossog B Ndo C Menze-Djantio B Zebaze-Togouet S Awono-Ambene H Costantini C Wondji C Ranson H Anopheles gambiae distribution and insecticide resistance in the cities of Douala and Yaoundé (Cameroon): influence of urban agriculture and pollution Malar J 2011 10 154 10.1186/1475-2875-10-154 21651761 PMC3118161

[B11] BUCREP Troisième recencement générale de la population et de l'habitat. Third general population and housing census Cameroun Rapport de présentation des résulstats définitifs République du Cameroun 2010

[B12] Gillies M Coetzee M A supplement to the Anophelinae of Africa south of the Sahara (Afrotropical region) Pub South Afr Inst Med Res 1987

[B13] Gillies M De Meillon B The Anophelinae of Africa South of the Sahara 1968 South Africa Institute of Medical Research Johannesburg, South Africa

[B14] Fanello C Santolamazza F Della Torre A Simultaneous identification of species and molecular forms of the Anopheles gambiae complex by PCR-RFLP Med Vet Entomol 2002 16 461 464 10.1046/j.1365-2915.2002.00393.x 12510902

[B15] Wirtz R Zavala F Charoenvit Y Campbell G Burkot T Schneider I Esser K Beaudoin R Andre R Comparative testing of monoclonal antibodies against Plasmodium falciparum sporozoites for ELISA development Bull World Health Organ 1987 65 39 45 3555879 PMC2490858

[B16] Abbott WS A method of computing the effectiveness of an insecticide J Eco Entomol 1925 18 265 267 3333059

[B17] WHO Test procedures for insecticide resistance monitoring in malaria Vectors. Bio-efficacy and Persistence of insecticides on treated surfaces WHO/MAL/98,12 Report of the WHO Informal Consultation, Geneva 1998

[B18] Lynd A Ranson H McCall P Randle N Black W Walker E Donnelly M A simplified high-throughput method for pyrethroid knock-down resistance (kdr) detection in Anopheles gambiae Malar J 2005 4 16 10.1186/1475-2875-4-16 15766386 PMC555548

[B19] Overgaard H Saebo S Reddy M Reddy V Abaga S Matias A Slotman M Light traps fail to estimate reliable malaria mosquito biting rates on Bioko Island, Equatorial Guinea Malar J 2012 11 1 56 10.1186/1475-2875-11-56 22364588 PMC3384454

[B20] Mourou J Coffinet T Jarjaval F Pradines B Amalvict R Rogier C Kombila M Pages F Malaria transmission and insecticide resistance of Anopheles gambia in Libreville and Port-Gentil, Gabon Malar J 2010 9 321 10.1186/1475-2875-9-321 21070655 PMC2995799

[B21] Mourou J-R Coffinet T Jarjaval F Cotteaux C Pradines E Godefroy L Kombila M Pages F Malaria transmission in Libreville: results of a one year survey Malar J 2012 11 1 40 10.1186/1475-2875-11-40 22321336 PMC3310827

[B22] Machault V Gadiaga L Vignolles C Jarjaval F Bouzid S Sokhna C Lacaux J Trape J Rogier C Pages F Highly focused anopheline breeding sites and malaria transmission in Dakar Malar J 2009 8 138 10.1186/1475-2875-8-138 19552809 PMC2713260

[B23] Klinkenberg E McCall P Wilson M Amerasinghe F Donnelly M Impact of urban agriculture on malaria vectors in Accra, Ghana Malar J 2008 7 151 10.1186/1475-2875-7-151 18680565 PMC2515328

[B24] Klinkenberg E McCall P Hastings I Wilson M Amerasinghe F Donnelly M High malaria prevalence and urban agriculture in Accra, Ghana Emerg Infect Dis 2005 11 1290 1293 16102322 10.3201/eid1108.041095 PMC3320508

[B25] Afrane Y Klinkenberg E Drechsel P Owusu-Daaku K Garms R Kruppa T Does irrigated urban agriculture influence the transmission of malaria in the city of Kumasi, Ghana? Acta Trop 2004 89 125 134 10.1016/j.actatropica.2003.06.001 14732235

[B26] Sattler M Mtasiwa D Kiama M Premji Z Tanner M Killeen G Lengeler C Habitat characterization and spatial distribution of Anopheles sp. mosquito larvae in Dar es Salaam (Tanzania) during an extended dry period Malar J 2005 4 4 10.1186/1475-2875-4-4 15649333 PMC546229

[B27] Manga L Robert V Messi J Desfontaines M Carnevale P Le paludisme urbain à Yaoundé, Cameroun. 1- Etude entomologique dans deux quartiers centraux Mém Soc R Belge Entomol 1992 35 155 162

[B28] Simard F Ayala D Kamdem G Pombi M Etouna J Ose K Fotsing J Fontenille D Besansky N Costantini C Ecological niche partitioning between Anopheles gambiae molecular forms in Cameroon: the ecological side of speciation BMC Ecol 2009 9 17 10.1186/1472-6785-9-17 19460146 PMC2698860

[B29] Bigoga JD Manga L Titanji VPK Etang J Coetzee M Leke RGF Susceptibility of Anopheles gambiae Giles (Diptera: Culicidae) to pyrethroids, DDT and carbosulfan in coastal Cameroon Afr Entomol 2007 15 1 1 7 10.4001/1021-3589-15.1.1

[B30] Ndjemai H Patchoke S Atangana J Etang J Simard F Bilong Bilong C Reimer L Cornel A Lanzaro C Fondjo E The distribution of insecticide resistance in Anopheles gambiae s.l populations from Cameroon: an update Trans R Soc Trop Med Hyg 2008 103 1127 1138 19155034 10.1016/j.trstmh.2008.11.018

[B31] Wondji C Frederic S Petrarca V Etang J Santolamazza F Della Torre A Fontenille D Species and populations of the Anopheles gambiae complex in Cameroon with special emphasis on chromosomal and molecular forms of Anopheles gambiae s.s J Med Entomol 2005 42 998 1005 10.1603/0022-2585(2005)042[0998:SAPOTA]2.0.CO;2 16465741

[B32] Dia I Diallo D Duchemin J Ba Y Konate L Costantini C Diallo M Comparisons of Human-Landing Catches and Odor-Baited Entry Traps for Sampling Malaria Vectors in Senegal J Med Entomol 2005 42 104 109 10.1603/0022-2585(2005)042[0104:COHCAO]2.0.CO;2 15799517

[B33] Kweka E Mahande A Comparative evaluation of four mosquitoes sampling methods in rice irrigation schemes of lower Moshi, northern Tanzania Malar J 2009 8 149 10.1186/1475-2875-8-149 19580663 PMC2712478

[B34] Govella N Chaki P Geissbuhler Y Kannady K Okumu F Charlwood J Anderson R Killeen G A new tent trap for sampling exophagic and endophagic members of the Anopheles gambiae complex Malar J 2009 8 157 10.1186/1475-2875-8-157 19602253 PMC2720981

[B35] Govella N Chaki P Mpangile J Killeen G Monitoring mosquitoes in urban Dar es Salaam: Evaluation of resting boxes, window exit traps, CDC light traps, Ifakara tent traps and human landing catches Parasites Vectors 2011 4 1 40 10.1186/1756-3305-4-40 21418622 PMC3069960

[B36] Trape J Lefebvre-Zante E Legros FGN Bouganali H Druilhe P Salem G Vector density gradients and the epidemiology of urban malaria in Dakar, Senegal AmJTrop Med Hyg 1992 47 2 181 189 10.4269/ajtmh.1992.47.181 1354414

[B37] Robert V Macintyre K Keating J Trape J Duchemin J Warren M Beier J Malaria transmission in urban sub-saharan Africa AmJTrop Med Hyg 2003 68 169 176 12641407

[B38] Pankoui-Mfonkeu J Gouado I Fotso-Kuate H Zambou O Grau G Combes V Amvam-Zollo P Clinical Presentation, Haematological Indices and Management of Children with Severe and Uncomplicated Malaria in Douala, Cameroon Pak J Biol Sci 2008 11 2401 2406 10.3923/pjbs.2008.2401.2406 19137849

[B39] McGuinness D Koram K Bennett S Wagner G Nkrumah F Riley E Clinical case definitions for malaria: clinical malaria associated with very low parasite densities in African infants Trans R Soc Trop Med Hyg 1998 92 5 527 531 10.1016/S0035-9203(98)90902-6 9861370

[B40] Koram K Bennett S Adiamah J Greenwood B Socio-economic risk factors for malaria in a peri-urban area of The Gambia Trans R Soc Trop Med Hyg 1995 89 2 146 150 10.1016/0035-9203(95)90471-9 7778137

[B41] Koram K Owusu-Agyei S Fryauff D Anto F Atuguba F Hodgson A Hoffman S Nkrumah F Seasonal profiles of malaria infection, anaemia, and bednet use among age groups and communities in northern Ghana Trop Med Int Health 2003 8 793 802 10.1046/j.1365-3156.2003.01092.x 12950665

[B42] Klinkenberg E McCall P Wilson M Akoto A Amerasinghe F Bates I Verhoeff F Barnish G Donnelly M Urban malaria and anaemia in children: a cross-sectional survey in two cities of Ghana Trop Med Int Health 2006 11 578 588 10.1111/j.1365-3156.2006.01609.x 16640609

[B43] Etang J Manga L Toto J Guillet P Fondjo E Chandre F Spectrum of metabolic-based resistance to DDT and pyrethroids in Anopheles gambiae s.l populations from Cameroon J Vect Ecol 2007 32 1 123 133 10.3376/1081-1710(2007)32[123:SOMRTD]2.0.CO;2 17633433

[B44] Ndo C Menze-Djantio B Antonio-Nkondjio C Awareness, attitudes and prevention of malaria in the cities of Douala and Yaoudé (Cameroon) Parasites Vectors 2011 4 181 10.1186/1756-3305-4-181 21933411 PMC3192766

[B45] Nwane P Etang J Chouaibou M Toto J Kerah-Hinzoumbe C Mimpfoundi R Awono-Ambene H Simard F Trends in DDT and pyrethroid resistance in Anopheles gambiae s.s. populations from urban and agro-industrial settings in southern Cameroon BMC Infect Dis 2009 9 163 10.1186/1471-2334-9-163 19793389 PMC2764715

[B46] Yadouleton A Asidi A Djouaka R Braima J Agossou C Akogbeto M Development of vegetable farming: a cause of the emergence of insecticide resistance in populations of Anopheles gambiae in urban areas of Benin Malar J 2009 8 103 10.1186/1475-2875-8-103 19442297 PMC2686728

[B47] N’Guessan R Corbel V Akogbeto M Rowland M Reduced efficacy of insecticide-treated nets and indoor residual spraying for malaria control in pyrethroid resistance area, Benin Emerg Infect Dis 2007 13 199 206 10.3201/eid1302.060631 17479880 PMC2725864

[B48] Corbel V Chabi J Dabire R Etang J Nwane P Pigeon O Akogbeto M Hougard J Field efficacy of a new mosaic long-lasting mosquito net (PermaNet 3.0) against pyrethroid-resistant malaria vectors: a multi centre study in Western and Central Africa Malar J 2010 9 113 10.1186/1475-2875-9-113 20423479 PMC2877060

